# Challenges in healthcare facilities’ response to past outbreaks: a systematic review of reviews

**DOI:** 10.1186/s12913-025-13934-9

**Published:** 2026-01-23

**Authors:** Elena Rubini, Monica Trentin, Paola Maffi, Bassma Aammar, Stanislav Gaievskyi, Awsan Bahattab, Hamdi Lamine, Maryam Kordi-Kaiser, Kaspar Staub, Luca Ragazzoni

**Affiliations:** 1 CRIMEDIM – Center for Research and Training in Disaster Medicine, Humanitarian Aid and Global Health, Università del Piemonte Orientale, 28100 Novara, Italy; 2 Department for Sustainable Development and Ecological Transition, Università del Piemonte Orientale, 13100 Vercelli, Italy; 3 Community Health Nursing, College of Nursing, University of Hail, Hail, 55424 Saudi Arabia; 4 Institute of Evolutionary Medicine, University of Zurich, Zurich, 8057 Switzerland; 5 Crisis Competence Center (CCC), University of Zurich, Zurich, 8006 Switzerland

**Keywords:** COVID-19, Disease outbreaks, Health personnel, Pandemic preparedness, Epidemics, Healthcare facilities, Health system, Systematic review

## Abstract

**Introduction:**

The frequency of infectious diseases outbreaks is increasing, but these challenges are not always thoroughly investigated. To date, no systematic reviews of reviews have comprehensively mapped the challenges and gaps faced during past epidemics and pandemics in Europe at healthcare facility level. This systematic review of reviews aims at filling this gap and at contributing to documenting the challenges and informing policy recommendations.

**Methods:**

This review was conducted within the Horizon Europe project PREPSHIELD. The search was conducted in October 2024 on PubMed, Scopus, and Web of Science. Reviews published in English between the years 2009–2024, reporting data on COVID-19, H1N1, influenza or seasonal flu, measles, or Mpox, and documenting gaps or challenges in the response to these outbreaks at healthcare facility level were included.

**Results:**

A total of 21 reviews were included: they were systematic, scoping, narrative, rapid, or integrative. The studies included in the reviews were mostly conducted in the United Kingdom, Italy, Spain, and France. The studies included different levels of healthcare facilities, namely hospitals, primary healthcare, and prehospital settings. Findings were classified according to the 4S surge capacity framework and were related to staff (*n* = 18), stuff (*n* = 14), space (*n* = 7), and system (*n* = 18). The most reported challenges in the included reviews were increased workload, mental health, and task shifting or redeployment, resource shortages, intensive care unit/emergency department space limitations and repurposing, and telemedicine challenges.

**Conclusion:**

Key issues described highlight the need for training, peer-to-peer support mechanisms, and improved inter-institutional collaboration. Technology was described as both an opportunity and a challenge in pandemic response if not adequately supported by health staff training. A whole-of-society strategy, combining all-hazards and hazard-specific approaches, and moving beyond crisis mode, can strengthen future outbreak preparedness and response.

**Supplementary Information:**

The online version contains supplementary material available at 10.1186/s12913-025-13934-9.

## Introduction

Over the past century, Europe has experienced several infectious disease outbreaks [1]. More recently, the Coronavirus disease 2019 (COVID-19) pandemic placed unprecedented strain on healthcare systems globally, highlighting systemic vulnerabilities and gaps in preparedness. In 2024, a disease prioritization process led by the World Health Organization (WHO) identified 34 priority pathogens with a high risk of causing a public health emergency of international concern [2]. The year 2024 recorded the highest measles incidence in 25 years [3]. Mpox was similarly identified as a health threat in a recent outbreak in Europe. The frequency and intensity of zoonoses and other disease outbreaks are projected to increase in the coming years, driven and amplified by the combined effects of climate change and globalization [4–7]. Addressing these complex challenges requires a coordinated system-wide response that fully engages the entire health system [8]. 

To enable an effective and resilient response to future health emergencies, it is essential to systematically analyze past experiences, transforming encountered challenges into actionable lessons learned [9] aligning with recommendations from the WHO and embodied in the After-Action Review methodology [10]. Research may play a key role in this process by systematically examining past response efforts, uncovering specific gaps and successful interventions, and generating context-sensitive evidence to guide policy and decision-making across diverse settings [11]. 

After the start of the COVID-19 pandemic, many reviews of the literature have been published due to the increase in the number of publications in that period and the necessity to synthesize emerging knowledge [12]. Some of them have attempted to systematically identify and categorize the challenges experienced at the healthcare facility level to inform strategies to “build back better” and enhance system resilience against future health crises [13–16]. To the best of our knowledge, there are no systematic reviews of reviews focusing specifically on the European contexts, and a comprehensive mapping of challenges provoked by infectious diseases outbreaks has not yet been published. To address this gap, we conducted a systematic review of reviews to synthesize a wide range of data and insights, capturing variations across European countries, healthcare systems, and professional roles. The research question that guided our study was “What evidence exists in literature reviews regarding gaps in healthcare facilities’ preparedness and response during past infectious disease outbreaks in Europe?”. By answering this question, we aim to generate insights that can inform future policy and practical interventions, ultimately supporting more coordinated and effective responses to infectious diseases outbreaks at the European level. In addition, we seek to provide a descriptive groundwork for future, more targeted studies in this field.

## Methods

A systematic review of reviews reporting evidence on gaps in healthcare facilities’ response to previous outbreaks in Europe was conducted. Our intent was not to provide an evaluation of evidence strength, but rather to contribute with a descriptive, exploratory mapping of challenges experienced at healthcare facility level. This review is part of the European Union’s Horizon Europe Project PREPSHIELD (Preparedness for Society in Health Crises and Disasters) project (n°101168124) funded under ‘HORIZONCL3-2023-DRS-01-01, *Improving Social and Societal Preparedness for Disaster Response and Health Emergencies*’.

### Data sources and search strategy

This review was guided by the Joanna Briggs Institute methodology for systematic reviews [17]. The Preferred Reporting Items for Systematic Reviews and Meta-Analyses (PRISMA) 2020 checklist [18] was used to report each stage of the review and its findings. Data presented here also adheres to the PRISMA-S extension for reporting systematic review search methods [19].

The search string (Additional file 1) included three blocks of terms related to outbreaks, healthcare facilities, and gaps. Filters used were “systematic review” and “review”, “English”, and for years “2009–2024”. The search was conducted on October 10, 2024, on three databases (PubMed, Scopus, and Web of Science).

After removal of duplicates, a pilot screening was conducted to ensure consistency in the application of the eligibility criteria. Then, titles and abstracts were manually screened by six investigators (ER, MT, BA, SG, AB, HL), followed by a full text review performed by two co-authors (PM, BA). In all phases, discrepancies were solved after discussion.

### Operational definitions

For the purpose of the present study, the following operational definitions were employed:

- Healthcare facility: it encompassed hospitals (general and specialized, district or first-level referral hospitals, including pharmacies situated within hospitals), primary healthcare (PHC) facilities, and prehospital units.
- Gaps or challenges in the response: they were conceptualized as only related to the response to outbreaks at the healthcare facility level. Namely, challenges solely provoked by the pathogen itself and not related to its management were not included.

### Eligibility criteria

Studies were included when they: (a) were reviews; (b) were published in English between the years 2009–2024 (time frame chosen to include studies focusing on H1N1); (c) reported data on COVID-19, H1N1, influenza or seasonal flu, measles, or Mpox – the most recent impactful outbreaks; (d) documented data from Europe; (e) described gaps or challenges in the response to these outbreaks at the healthcare facility level. Studies were excluded when they did not meet eligibility criteria or when it was not possible to extract data only concerning the outbreaks and European countries mentioned.

### Data extraction, analysis, and reporting

A Google Sheet was developed to extract relevant data from the eligible articles (Additional file 2), including information on the study and study design, about the outbreak, healthcare facility, and gap in the response. Data was extracted by two investigators (PM, BA) who independently performed the coding and theme development. A team of four researchers analyzed the data using a deductive thematic approach guided by the 4S framework (staff, stuff, space, system) (Table 1). Extracted findings were coded against each of the four dimensions, refined through iterative comparison, and synthesized into overarching themes. This enabled systematic interpretation of the data, while maintaining coherence and consistency. Any discrepancies, or instances in which studies could be assigned to multiple categories, were resolved through team discussion to reach consensus on the classification that best reflected the nature of each challenge. Findings are presented according to the 4S framework, emphasizing the key thematic patterns identified through the analysis.

**Table 1:** Operational definitions for the components of the 4S framework

Component	Definition
Staff	Personnel involved in healthcare delivery and hospital operations.
Stuff	Physical equipment required to deliver healthcare and support healthcare delivery.
System	Planning and leadership activities implemented to operationalize and optimize a response effort. For the purpose of the present study, “system” entails different aspects such as guidelines, clinical protocols, training, communication, and patient-centered care (e.g., introduction of telemedicine and new procedures for managing and treating patients within healthcare facilities).
Space	Physical spaces for patient care.

## Results

**Fig. 1:**
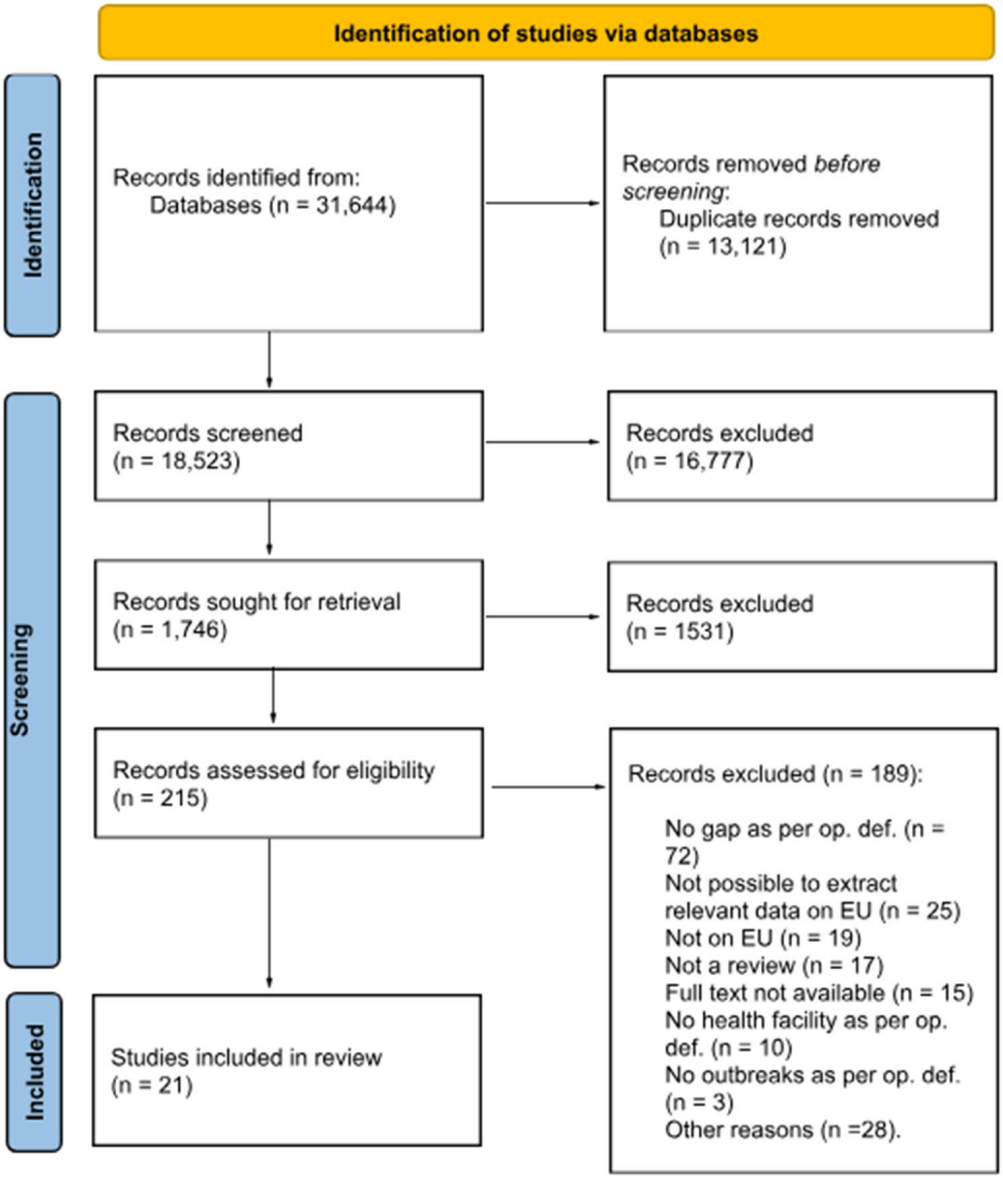
Study selection process

Detailed information on the study selection process can be found in the PRISMA diagram (Fig. 1), while Table 2 shows the main characteristics of the eligible studies.

**Table 2:** Characteristics of included studies

	Author / Year	Country where the included studies were conducted	Study type	Search period	Study objective	Outbreak type and pathogen mentioned as per the operational definition
1.	Cadel et al. (2021) [20]	France, Italy, Sweden, Türkiye, United Kingdom (UK)	Scoping Review	March 11–September 1, 2020	To identify the broad range of patient engagement activities within health systems during the first six months of the COVID-19 pandemic, and the key barriers and facilitators to sustain these activities.	Pandemic; COVID-19
2.	Hamid et al. (2023) [21]	France, Spain, Türkiye	Systematic Review	2019–2022	The impact of the 2019 COVID-19 season on intensive care units (ICU) work.	Pandemic; COVID-19
3.	Ong et al. (2021) [22]	France, Germany, Italy	Narrative Review	January–August 2020	To describe the changes in out-of-hospital cardiac arrest characteristics in several areas during the COVID-19 pandemic, and to review the logistical challenges and exposure risks faced by first responders managing prehospital sudden cardiac events during COVID-19.	Pandemic; COVID-19
4.	Aydogdu (2023) [16]	Denmark, Iceland, Spain	Integrative Review	2019–2022	To identify the challenges faced by nurse managers during the COVID-19 pandemic.	Pandemic; COVID-19
5.	Pujolar et al. (2022) [23]	Belgium, Germany, Greece, Ireland, Italy, Slovenia, Spain, Switzerland, Türkiye, UK	Scoping Review	December 2019–September 2021	To synthesize the knowledge accrued since the onset of the COVID-19 pandemic regarding its impact on access to healthcare services for non-COVID-related conditions, and to identify knowledge gaps on these topics.	Pandemic; COVID-19
6.	Lin et al. (2022) [24]	Italy, Türkiye	Scoping Review	From inception to February 2022	To explore the relevant evidence about stress-related cognitive appraisal and coping strategies among registered nurses dealing with the COVID-19 pandemic in the emergency department.	Pandemic; COVID-19
7.	Holthof et al. (2021) [25]	Italy, UK	Rapid Review	February–November 2020	To review the lessons learned from previous infectious disease outbreaks and the current COVID-19 pandemic. To discuss the implications of pandemic work for the staff’s physical and mental health, and provide ideas for limiting staff shortages and creating surge capacity in acute care.	Pandemic; COVID-19
8.	Katz et al. (2020) [26]	Italy, Spain, UK	Rapid Review	Not specified	To offer pragmatic suggestions on how to implement scalable models for critical care delivery, cultivate educational tools for team training, and embrace technologies to enable effective collaboration despite social distancing imperatives.	Pandemic; COVID-19
9.	Matenge et al. (2022) [27]	Belgium, France, UK	Systematic Review	From inception to December 2020	To identify and summarise practice-level strategies used to ensure the provision of routine primary healthcare during the COVID-19 pandemic response to inform current and future practice.	Pandemic; COVID-19
10.	Alsahli et al. (2023) [28]	Germany	Systematic Review	March 2020–December 2022	To explore and synthesize the scientific literature on the factors influencing the acceptance and adoption of mobile health (mHealth) apps among physicians during the COVID-19 pandemic.	Pandemic; COVID-19
11.	He et al. (2021) [29]	Crete (Greece), Czech Republic, Ireland, Italy, Romania, UK	Scoping Review	2011–2020	To identify key cybersecurity challenges, solutions adapted by the health sector, and areas to improve against recent cyberattacks that exploit vulnerabilities introduced by changes to working practices in response to the COVID-19 pandemic.	Pandemic; COVID-19
12.	Hill et al. (2023) [15]	France, Spain, The Netherlands, UK	Scoping Review	December 2019–May 2021	To provide an overview of PICU (pediatric intensive care units) care organisation during the first 18 months of the COVID-19 pandemic. It categorized evidence, mapped existing studies, and explored PICU organisation during that period.	Pandemic; COVID-19
13.	Kissel et al. (2023) [30]	Belgium, France, Germany, Spain, Sweden, The Netherlands, Türkiye, UK, Ukraine	Scoping Review	2020–January 2022	To identify the impact of the COVID-19 pandemic on nurses working in ICU.	Pandemic; COVID-19
14.	Zangani et al. (2022) [31]	Austria, Czech Republic, France, Germany, Greece, Italy, Spain, Slovakia, UK	Systematic Review	March 2020– April 2021	To understand the degree to which service provision has changed during the first year of the pandemic and the extent of the transition to tele mental health in different countries.	Pandemic; COVID-19
15.	Fernandez et al. (2020) [32]	UK	Systematic Review	Not specified	To synthesize and present current evidence around nurses’ managing and caring for patients during pandemics.	Pandemic; Influenza
16.	Ng et al. (2022) [33]	Belgium, France, Germany, Poland, Portugal, UK	Systematic Review	From inception to July 2021	To systematically examine the pattern of COVID-19 outbreaks in hospitals and identify vulnerable aspects to mitigate the risk of infection.	Pandemic; COVID-19
17.	Botma and Roets (2023) [34]	Italy, Spain, UK	Systematic Review	December 2019–February 2021	To interpret qualitative findings across multiple studies worldwide to delineate the influences on the mental health of healthcare professionals during the COVID-19 pandemic and deduce the impact on managerial responsibilities.	Pandemic; COVID-19
18.	Sellers et al. (2024) [35]	Czech Republic, France, Spain, Türkiye, UK	Systematic Review	From inception to September 2022	To explore the practical considerations of implementing crisis standards care in ICU during patient surges resulting from disaster events.	Pandemic; COVID-19
19.	Hamis et al. (2023) [14]	Italy, Romania, Spain	Scoping Review	January 2020–December 2021	To review the relevant literature on COVID-19 field hospital implementation strategies, challenges and opportunities.	Pandemic COVID-19
20.	Kirienko et al. (2021) [36]	Europe	Narrative Review	From inception until January 21, 2021	To determine and describe the impact of COVID-19 on nuclear medicine in Europe and to critically discuss the actions and strategies applied to face the pandemic.	Pandemic; COVID-19
21.	Rourke et al. (2023) [13]	France, Spain, Sweden, Türkiye, UK	Scoping Review	Not specified.	To critically synthesize the qualitative literature to understand the experiences of critical care nurses during the COVID-19 pandemic.	Pandemic COVID-19

### Characteristics of the studies

All but one, who dealt with the 2009 influenza pandemic [32], of the included studies focused on the COVID-19 pandemic and were published between 2020 and 2023.

**Fig. 2:**
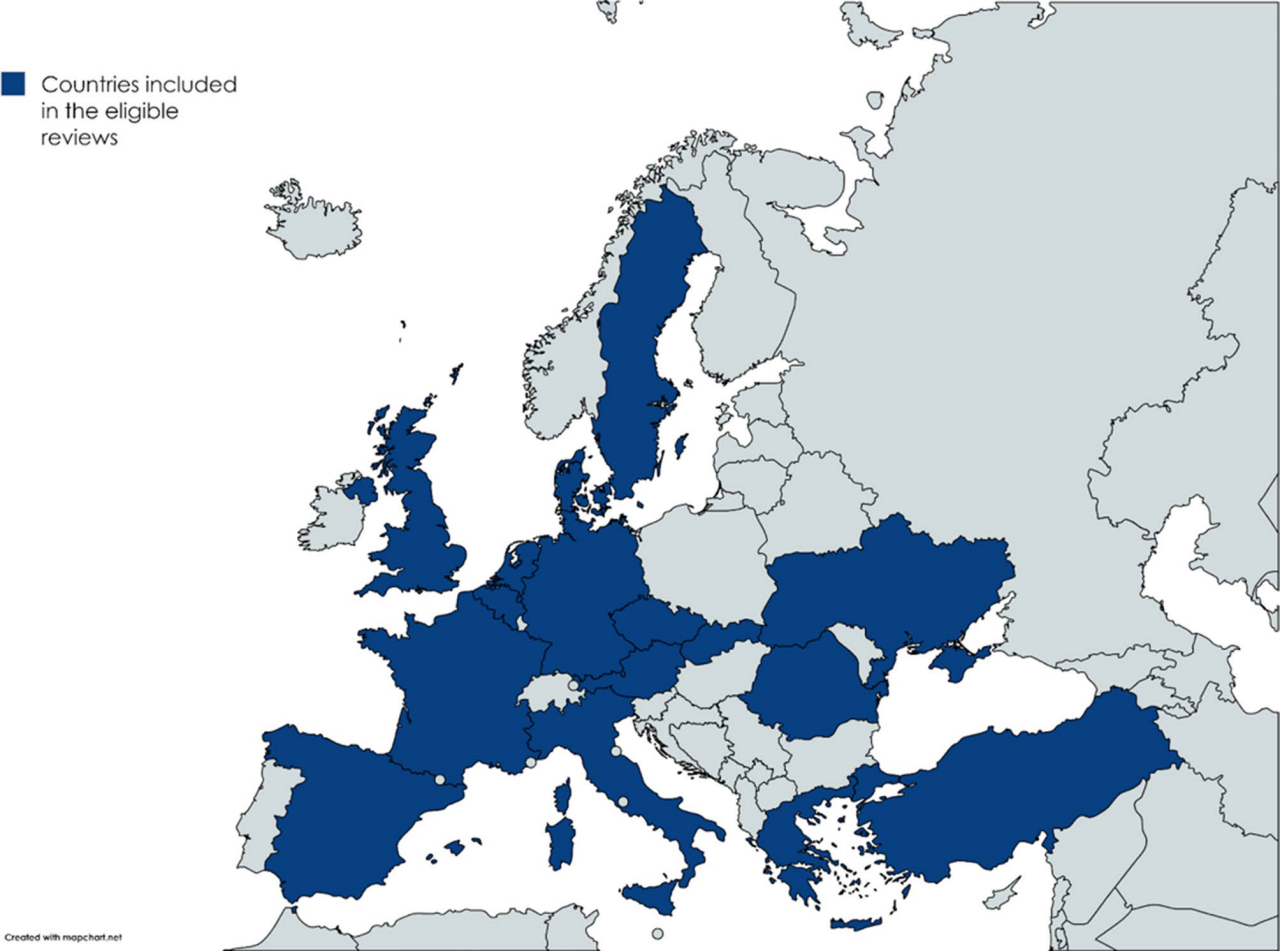
Map showing the countries where the studies included in the eligible reviews were conducted

As for the study design, reviews were systematic (*n* = 8) [13, 21, 27, 28, 31–34], scoping (*n* = 8) [14, 15, 20, 23, 24, 29, 30, 35], narrative (*n* = 2) [22, 36], rapid (*n* = 2) [25, 26], or integrative (*n* = 1) [16]. Most frequently, the studies included in the reviews were conducted in the United Kingdom (UK) (*n* = 16) [13, 15, 20, 23, 25–27, 29–35], Italy (*n* = 11) [14, 15, 20, 22–26, 29, 31, 34], Spain (*n* = 10) [13, 15, 16, 21, 23, 26, 30, 31, 34, 35], and France (*n* = 10) [13, 15, 20–22, 27, 30, 31, 33, 35]. An overview of the countries covered by the eligible reviews can be found in Fig. 2.

The studies included different levels of healthcare facilities, namely hospitals (*n* = 17) [13–16, 21–26, 29, 30, 32–36], including intensive care units (ICUs) (*n* = 7) [13, 15, 21, 25, 26, 30, 35] and emergency departments (EDs) (*n* = 3) [24, 32, 34], PHC (*n* = 4) [20, 23, 27, 31], and prehospital settings (*n* = 3) [22, 34].

The study objective revolved around the impact of COVID-19 on health-related working activities (*n* = 9) [13, 14, 16, 21, 22, 31, 32, 35, 36], best practices and lessons learned (*n* = 6) [15, 23, 25–27, 33], technology (*n* = 2) [28, 29], mental health of staff (*n* = 3) [24, 30, 34], the impact of influenza on working activities (*n* = 1) [32], and patient engagement (*n* = 1) [20].

### Healthcare facility challenges classified according to the 4S framework

This Section will present the challenges and gaps in healthcare facilities’ response to previous outbreaks, along with adaptation strategies, best practices, and lessons learned, based on the 4S framework (staff, stuff, space, system). Although described separately, these components interact and exacerbate their impacts.

#### Staff

The component “Staff” (*n* = 16) [13–16, 21–25, 28–30, 32, 34–36] revolved around several sub-themes, namely “specialized staff”, “task shifting or redeployment”, “increased workload”, “personal protective equipment (PPE) usage”, “mental health”, “relationship between patient and health provider”, “suboptimal standards of care”, “medicolegal concerns” and “telemedicine technologies”.

A lack or limited availability of specialized critical care staff, also due to healthcare workers (HCWs) becoming infected with COVID-19, their pregnant or lactating status, and other conditions [16] was described [14, 16, 25–27]. To address these challenges, several strategies were implemented. In some cases, enlarging the number of critical care nursing staff was described as a best practice [24]. In others, task shifting or redeployment were used to overcome shortages of specialized staff, or to manage increased workload, although these measures were often described as challenging [13–15, 24, 27, 30, 34–36]. These adjustments frequently resulted in changes to nurses’ roles and responsibilities [37], with many being reassigned to unfamiliar working environments [30]. Redeployed staff reported feeling unprepared for performing specific clinical tasks [30, 34]. Pediatric ICU staff reassigned to adult wards [15] faced clinical difficulties due to differences in anatomy, symptoms presentation, comorbidities, and treatment of adult patients, which required ICU staff to oversee their work to ensure standards of care were met [15]. Despite the difficulties, collaborative multidisciplinary teams enabled non-ICU staff redeployment [15, 30, 35] and proved to be an efficient strategy in addressing high patient volume [30]. 

Increased workload compared to pre-pandemic times was also vastly reported [14, 16, 21–23, 28, 30]. This led to longer COVID-19 patient contact times, greater exposure to work-related health hazards, and reduced availability of staff to care for non-COVID patients [21, 23]. At times, increased workload was correlated with staff shortages [14, 24], especially in critical care settings [24]. This, together with required overtime or long working hours and high complex-needs patient volume, was a risk factor for burnout among health staff [30]. 

PPE dressing and undressing procedures were described as “exhausting” [13, 24], but also as a form of teamwork and a “social ritual” with the routine of donning and doffing usually being conducted in pairs to both check PPE fit and each other’s mental health [13]. Conversely, lack of PPE resulted in burnout [30], fear [31], and heightened stress among healthcare professionals [24].

Some reviews [13, 15, 16, 21, 24, 30, 32, 34, 36] reported the consequences of working during COVID-19 or influenza pandemics on health staff mental health. These encompassed stress, depression, anxiety, fear (including fear of dying), worries, and burnout, which could lead them to quitting their job [16, 21, 30]. At times, the quick transition from one ward to another, as well as the lack of recognition for their efforts from broader society, worsened these mental health symptoms. Uncertainty and “tumbling into chaos” [13, 30] as related to HCWs’ own health, that of their patients, the uncertainty surrounding how long the peak of infections would last, and the inability to offer care meeting the usual standards were identified as key elements in aggravating mental health [30]. Health staff reported negative feelings due to surges in cases, witnessing other colleagues getting infected and becoming ill or dying, and being physically aggressed by visitors; they often complained of not receiving support from their managers. This had a cascading effect on their working activities, leading to an inability to work or compromising care [13, 24, 35, 36]. Despite the challenges encountered, many reviews described positive coping strategies. The concept of “care” was extended beyond their working activity to include closeness, protection and safety, teamwork, trust, cohesion, and mutual respect [13, 16, 24]. Psychological support, particularly peer monitoring, was fundamental for stress mitigation and improve post-shift decompression [24, 34]. Communication and support between team members were also critical, together with interdisciplinary collaboration and strong leadership in ensuring a good working environment [15, 30, 32, 34]. 

The relationship between patients and health staff, suboptimal standards of care, and medicolegal concerns impacted patient care and communication. The relationship between patients and health providers was described [13, 15, 16, 24, 30, 34], noting how healthcare staff became more aware of their role as a “bridge” facilitating communication between patients and families [16]. However, their interactions with patients often became robotic and depersonalized [30, 34]. Health staff were concerned patient isolation could hinder care [30]. Creating meaningful and effective interactions with them, even those who were at high risk or in isolation, and their family members, was described as best practices [13, 15, 24]. However, the use of PPE led to communication problems caused by the loss of non-verbal communication [20]. 

Health providers [13, 16, 30] were concerned about the risk of offering suboptimal standards of care, especially during surges in cases of infection, as well as by fear and anxiety induced by their working environment magnifying this risk, and this had repercussions on their mental health [13, 30]. 

Health staff having medicolegal concerns due to the need to prioritize resources and patients’ needs during the pandemic was reported [28, 32], extending to diagnoses established through telemedicine or mobile apps, which were perceived as less clinically accurated [28]. Telemedicine technologies also led to other issues [28, 29, 36]: some providers were concerned about suboptimal service delivery [28] and the extra time needed for consultations [36]. Insufficient knowledge of those tools and of cybersecurity issues also hindered their use [28, 29]. 

#### Stuff

A total of 14 reviews examined the challenges associated with “Stuff” during the COVID-19 pandemic [14, 15, 22–24, 26–28, 30, 31, 35, 36], and/or adaptation strategies [13, 21, 24, 26, 35]. 

The concern reported in more reviews was the reduction in available resources such as PPE, medications, medical supplies, and ventilators [14, 15, 30, 35, 36]. These scarcities were particularly evident within hospital settings [23, 26, 31, 35, 36], especially in EDs [24] and ICUs [15, 30], but also observed in field hospitals [14], PHC [23, 27], and prehospital care settings [22]. 

The lack of PPE was widely reported [14, 24, 26, 27, 35, 36], at times leading to increasing pressure from local or national authorities to intervene and secure supplies that hospitals had traditionally sourced independently [26]. In other instances, ventilators were insufficient to meet the demands of a high volume of patients, and this shortage, compounded by other resource constraints, heavily influenced decision-making [35]. 

Resource constraints also forced hospitals to implement contingency measures, such as administering aseptically compounded medications beyond their expiration dates and reducing the frequency of intravenous tubing changes, deviating from standards of care [16]. Delays in the supply of radiopharmaceuticals during the COVID-19 pandemic were highlighted in a review on nuclear medicine [36].

To address resource shortages and optimize critical care capacity, strategies such as resource reallocation [21] and spatial reorganisation [15, 35] were employed [13]. Neonatal and portable ventilators were assigned to pediatric patients, allocating standard ventilators for adult use [35]. Additionally, several pediatric intensive care units (PICUs) adapted their layout to accommodate both child and adult patients within the same space, or transitioned to exclusively treating adults [15, 35]. While positive experiences in treating adults within PICUs were reported, this approach also presented several challenges connected to redistribution of equipment and supplies [15]. 

The shortage of materials and supplies adversely affected the delivery of non-COVID-related healthcare services across PHC, outpatient secondary care, and inpatient care [23]. The limited availability of technology and the lack of technical support were identified as the main barriers for its implementation [28]. Additionally, pandemic-related restrictions affected prehospital emergency services by limiting access to automatic external defibrillator stations. As a result, for instance, the Paris Fire Brigade had to revise out-of-hospital cardiac arrest protocols and update the rescue chain [22]. 

#### Space

A total of seven reviews addressed the theme of “Space” [14, 15, 24–26, 30, 35]. A challenge commonly described was the persistent space limitation to accommodate the surge in patients, particularly in ICUs and EDs. The issue was reported during the COVID-19 pandemic in the UK, France, Spain, Türkiye, and the Czech Republic [30, 35]. Facilities were forced to restrict the number of available ICUs beds due to equipment limitations [35] and this shortage forced healthcare facilities to repurpose non-ICU hospital spaces, cardiac ICUs, and specialized wards to provide ICU-level care [26]. Efforts to create new ICU spaces raised significant engineering concerns regarding the feasibility of establishing airborne isolation rooms and equipping these spaces with essential medical infrastructure, such as medical gas, vacuum systems, and emergency power outlets [26]. 

Another major concern was the inadequate separation of patients with different risk levels [24]. Space constraints were extended beyond patient care areas. Common spaces, such as break rooms and corridors, posed challenges in maintaining infection control [25]. Moreover, HCWs struggled with inadequate separation between “clean” and “dirty” areas [24]. 

In response to these space-related challenges, healthcare facilities demonstrated adaptability, repurposing clinical and non-clinical spaces, such as conference rooms and gyms, into functional patient care areas to address the critical shortage of beds [35]. New donning and doffing stations were created [15]. The conversion and expansion of PICUs into adult ICUs constituted both an opportunity and a challenge, as they required extensive modifications, major construction efforts, and coordinated multidisciplinary efforts [15]. These changes posed significant challenges in ensuring patient safety and adapting operational structures [15]. 

To further manage patient overflow, some countries converted existing public spaces such as exhibition halls and basketball courts into temporary field hospitals [14]. While these makeshift facilities were a cost-effective solution, they presented considerable challenges in infection control due to infrastructure limitations. The high demand for isolation rooms further complicated patient management, with many facilities struggling to provide sufficient availability of adequate infrastructure to ensure effective containment of infectious diseases [14, 30]. 

#### System

System-related challenges and best practices, focusing on the subthemes of “clinical protocols”, “patient-centered care”, “training”, and “communication” were mentioned in 18 articles [13–16, 20, 22–24, 26–32, 34–36]. 

The lack of clear guidance, treatment protocols, and standards contributed to a high level of uncertainty among HCWs, also due to the difficulties in providing patients with specific advice related to COVID-19 or influenza [13, 24, 27, 28, 32, 34, 35]. Additional challenges were posed by the lack of widely accepted international guidance and processes for resuscitation or bystander cardiopulmonary resuscitation (CPR) of out-of-hospital cardiac arrest during the pandemic [22]. In addition, in some countries, rapid organisational changes, working conditions, and the absence of protocols for prolonged mass casualty events were also described as challenges [30]. 

Lastly, inconsistent or sparse communication due to the lack of clear guidance on COVID-19 or influenza was highlighted as a challenge by multiple studies [14, 30, 32, 35]. 

Nevertheless, different articles have pointed out how incentives, awards, logistical support, and open management by nurse managers were essential in overcoming these challenges [16]. Interdisciplinary decision-making and leadership were essential for improving governance [14, 35]. 

A clear chain of command, excellent leadership, and constant support were used to supply the limited resources needed to deliver care [14]. In the specific case of transforming PICUs into adult ICUs, the reported benefits were shared administration, management, resources, and supply chains [15]. 

In Italy, COVID-19 caused difficulties in the system, especially in field hospitals, because of the increase in daily admissions and decrease in discharges [14]. However, it was important to define their main work area and dividing them into sections according to functions [14].

Guidelines that mandated the use of PPE led to less empathetic and longer consultations [27]. Concerning out-of-hospital cardiac arrest rescue procedures, additional PPE dressing time influenced resuscitation protocols, besides leading to delays in the departure of the emergency medical service personnel [22].

Telemedicine introduced significant challenges in terms of workload [27, 28, 30], information flow and time management, quality of care [13, 23], and communication systems [20, 23, 27, 30]. Regarding the latter, issues were at the patient and provider level, specifically connected to translation, to the limited intercultural communication, or to patients’ technological illiteracy [20, 23, 27, 30]. Ethical concerns, privacy issues, data protection, and security were also reported as challenges revolving around the adoption of the new telemedicine mode [27, 28]. Moreover, the system was exposed to a variety of cyberattacks due to the technologies used. Furthermore, the quick transition to remote consultation without appropriate guidelines resulted in a lack of security awareness among medical personnel [29]. Regarding data security, the Information Commissioner’s Office in the UK established an information portal to help people and organisations safeguard data during the COVID-19 outbreak, and the National Health System (NHS) added guidance on how to work from home securely [29]. 

During periods of high service utilization, HCWs had to ensure risk reporting, record keeping, and data monitoring of patients, leading to increased burden. ICU flow sheets with remote hemodynamic monitoring for drug dosing and patient progress assessment were essential to limit unnecessary exposure to infected patients, thus inducing safe remote decision-making [26]. In the case of nuclear medicine, operational modifications such as telemedicine for follow-up visits were essential, along with other prioritization measures. These included rescheduling of low priority procedures, the correct use of PPE, social distancing, personal hygiene, and prompt identification of COVID-19 cases through the use of nuclear medicine machines [36]. 

Clear and open communication [16, 21, 24] were pivotal in overcoming the challenges of telemedicine’s use and collaboration among HCWs. In some ICUs, dedicated time for staff meetings and interdisciplinary communication increased situational awareness and facilitated updates with family members, especially during surges [35]. 

Adaptations to care models for conducting consultations through phone or video calls posed significant challenges regarding patient-centered care. Phone consultations made clinical decisions and acute psychological care more difficult due to the limited information obtainable, possibly leading to misdiagnosis and consequently to liabilities for mistreatment [27, 28]. The shift to telemedicine encompassed end-of-life care decisions due to the visitor restriction policies, advanced directives, and resuscitation decisions being discussed over the phone with family members, leading to dehumanized care [34]. The lack of a clear guide led HCWs to not being able to provide quality patient care [13, 32], while the changing mode of care also led to increased waiting times for patients [23]. 

Videoconferencing and permitting a single person to make a compassionate visit during end-of-life situations, particularly in pediatric settings, were describes as best practices to avoid “dehumanized care” [15].

Significant concerns arose regarding the accessibility of virtual care for patients living in poverty and precariously housed in terms of internet connection and devices [27].

Several studies emphasized that staff members had issues working in ICUs because of a lack of training in critical care and on the use of control measures and that PPE led to challenges in caring for patients [13]. In Iceland, nurse managers used guidelines and modified emergency plans as coping measures, for example, including distributing the experienced staff among different hospital shifts and training organisations [16]. For instance, workshops, virtual seminars and simulations, and continuing education, including on topics such as policies of infection control and how to interact with COVID-19 patients and their relatives, were organized [16, 24]. Social media were used by nurse managers for acquiring and transmitting knowledge to their staff [16]. 

In other cases, already-in-place training programs (e.g., Basic Life Support and CPR training for emergency medical service personnel) were put on hold because of the pandemic’s insurgence [22]. Psychological support and training were identified as necessary to equip nurses and physicians with adequate knowledge about the disease [21, 24] and as contributing to positive feelings and to ensuring their mental well-being [30, 31]. In order to minimize the moral burden on HCWs, a separate triage committee to prioritize ICU admissions and ad hoc trainings in ethical aspects of the pandemic were introduced [35]. Additionally, monitoring of nursing management during COVID-19 was established to ensure they performed their duties correctly [21]. 

## Discussion

We conducted a descriptive, exploratory mapping of challenges experienced at healthcare facility level in their response to past outbreaks in Europe through a systematic review of reviews. All but one of the included studies in the time frame considered focused on the COVID-19 pandemic, showing a gap for what concerns the investigation and documentation of challenges in the response to other outbreaks. This also implies that during the COVID-19 pandemic it was difficult to find evidence of how to effectively respond to an outbreak in peer-reviewed literature and to use it for guiding the response to the health crisis. Conversely, this review of reviews could serve to summarize the challenges encountered during past outbreaks. Its findings could possibly serve in interpandemic or interepidemic phases to guide preventive initiatives, starting from the elaboration of recommendations for preparedness that will be tested in the next phases of Horizon Europe project PREPSHIELD, aiming at enhancing social and societal preparedness to pandemics, including at healthcare facility level.

In our study, the 4S Framework–Staff, Stuff, Space, and System – allowed us to categorize the challenges faced by healthcare facilities during the pandemic into four key areas [38, 39]. 

Following the 4S Framework, challenges described were related to staff (*n* = 16), stuff (*n* = 14), space (*n* = 7), and system (*n* = 20). The most reported challenges in the included reviews were for staff increased workload, mental health, and task shifting or redeployment; for stuff resource shortages; for space ICU/ED space limitations and repurposing; and for system telemedicine challenges. System is often siloed to encompass only planning and leadership [40], whereas several critical elements are often left out, such as training of personnel, patient-centered care, and cross-sector collaboration [38]. These missing elements are essential for ensuring effective surge capacity. Therefore, even though the 4S Framework proved to be a useful tool for supporting disaster preparedness at different levels, a broader approach such as the one employed in the present review is necessary to ensure all critical components are considered [39].

The results show the limited availability of specialized staff, emphasizing the need to train staff from other specialties to be able to scale up. In this regard, just-in-time training and simulation have been described in the literature at the global level as effective in the education and training of staff during pandemics [25, 41–43]. Bottom-up, peer-to-peer initiatives have also been implemented to share educational materials and to provide mental health support strategies [44–46]. While these initiatives have been administered during the COVID-19 outbreak, these should ideally be established in ordinary times and be included in healthcare curricula [47–49]. Additionally, collaboration between institutions specializing in disaster medicine training and hospitals can enhance the development of these initiatives, ensuring that training and preparedness efforts are continuously integrated and adapted to evolving needs. In the included reviews, adaptability was identified as crucial for healthcare facilities during health crises, encompassing all the dimensions of the 4S framework, in adherence with the literature [50, 51]. 

Our findings also highlight the effect that mental health issues had on the working activities of HCWs. This sheds light on the need for addressing and including this component in preparedness training activities, which has been linked to improved mental health outcomes [52–55].

Technology was described as both an opportunity and a potential obstacle. While distance training and telemedicine were fundamental for ensuring continuity of care, distance practice posed challenges (e.g., ethical and medicolegal concerns, limited knowledge of the tools, cybersecurity issues). Training initiatives during ordinary times should also encompass technology use [49]. 

These challenges underscore the critical need for pandemic preparedness. While implementing these initiatives, it is important to leverage a cross-cutting all-hazards approach [56] and a vertical hazard-specific approach [57]. The first should aim to strengthen the underlying capacities that are useful for managing risk and responses for many types of emergencies. The second should focus on capacities that are needed to manage the risks and responses to specific hazards of concern. By addressing a broad range of risks simultaneously, this strategy strengthens the resilience of healthcare systems, facilitating a more agile response to diverse emergencies.

At the European level, various initiatives are being implemented to strengthen health crisis preparedness in response to the significant gaps in readiness exposed by the COVID-19 pandemic. The European Commission has been enhancing its role in preparedness planning and response through the establishment of the European Union Health Emergency Preparedness and Response Authority, which is responsible for horizon scanning of major health threats and potential medical countermeasures, funding research and development, supporting manufacturing capacity, and stockpiling key medical supplies and equipment [58, 59]. Initiatives to gather feedback on strategies for supporting medical countermeasures, engaging citizens, businesses, civil society organisations, and experts have also been launched [60]. These initiatives align with the principles of a whole-of-society approach [61, 62], which emphasizes the importance of engaging all sectors and levels of society–governmental and non-governmental actors, the private sector, civil society, academia, and communities–in both preparedness and response activities [63, 64]. Our analysis of the challenges identified them as being present across multiple levels of healthcare facilities. However, further research should investigate the roles and challenges faced by other sectors [65] and intersectoral collaboration during COVID-19 to inform future strategies. Notably, the deployment of Emergency Medical Teams significantly increased during the pandemic, highlighting their critical role in addressing surge capacity and ensuring continuity of care in strained health systems. Fostering a whole-of-society approach aligns with the core objective of the PREPSHIELD (‘Preparedness for Society in Health Crises and Disasters’) project – strengthening the whole-of-society approach to pandemic preparedness – funded under ‘HORIZONCL3-2023-DRS-01-01, *Improving Social and Societal Preparedness for Disaster Response and Health Emergencies*’.

In light of the increasing number of epidemics and pandemics, it is essential to integrate a global health perspective into both research and operational practices, grounded in a whole-of-society approach. Global health must move beyond crisis-driven solutions focused on protecting the Global North with little public interest when these events do not impact this context [60, 66–68]. On the contrary, the Global Health Security Agenda must adopt a preparedness system that recognizes health as a shared global responsibility, rooted in human rights and international solidarity, where each state has a duty in ensuring “health for all” and recognizing the interdependence across nations that occurred after globalisation, particularly in terms of pandemic management [66, 69, 70]. Asymmetrical approaches should be dismissed in favour of recognizing the equal responsibilities that all actors have at the international level and establishing global partnerships, ensuring that each country has the capacity to contain the pathogen in case of a new outbreak [66, 71, 72]. Therefore, pandemic preparedness should be conceptualized as a collective responsibility, moving beyond crisis mode [71]. 

In addition, increasing research capacity means also including the One Health and Planetary Health frameworks [73], which link human, animal, and environmental health to provide accessible, science-based information also targeting health staff and the general population [71]. As a sustainable, whole-of-government [74], and whole-of-society approach, the One Health approach aims to identify preparedness strategies for health emergencies involving multidisciplinary and multi-sector actors, enhancing coordination and shared responsibility, thus supporting global health strategies [75]. Thus, the systemic dimension proves once again to be crucial in pandemic preparedness and response, aiming at a multidisciplinary, multi-actor, and global approach to future outbreaks.

### Strengths and limitations

This review has important strengths. It is grounded in the 4S framework—a well-established and widely applied model in disaster medicine – which provided a comprehensive structure for organizing and interpreting the findings. We used a broader definition of system, capturing its complexity and including other critical elements in our analysis that are crucial for assuring efficient surge capacity in the event of pandemics and epidemics.

This study also has some limitations. First, the focus on the European region was connected to the scope of the PREPSHIELD project, limiting our analysis in terms of geographical span and outbreaks; at times, the geographical context of reference was collected under the umbrella term Europe, with data presented in aggregated form, limiting the depths of our findings. We recognize that the challenges reported are strongly influenced by context- and country-specific factors, including healthcare system organization, governance, resource availability, and sociocultural context. Analyzing these factors in detail would have gone beyond the intended scope of this study, which aimed to provide a broad mapping of challenges faced by healthcare facilities across Europe, and was not feasible given that the included reviews did not focus on country-specific associations or provide the level of detail required. We did not perform a formal methodological quality appraisal of the included reviews because our primary aim was to descriptively map reported challenges, as, despite their varying rigor, they could contribute valuable insights into the types of challenges encountered at healthcare facility level. In addition, the inclusion of diverse review types (systematic, scoping, narrative, rapid, integrative) would have required multiple, non-comparable appraisal tools. As a result, our synthesis should be interpreted as a thematic consolidation of reported challenges rather than a quality-weighted assessment of the underlying evidence. Details were provided on the type of studies and methodological aspects to enable the reader to understand which studies the results came from. Another limitation concerns the fact that we did not perform a formal assessment of overlapping of primary studies across the included reviews, an aspect that is important to consider in order to avoid potential overrepresentation of the same findings. However, we manually checked all references of the included reviews to assess whether the same study was included in multiple reviews and identified that nine primary studies appeared in two reviews each. Given the broad scope of the 21 reviews included in our study, we believe that this limited overlap is unlikely to have led to a significant overrepresentation of findings. Notably, our presentation of results was based on thematic patterns rather than on the frequency with which a specific challenge appeared across reviews. Lastly, including only studies published in English might have resulted in the exclusion of other relevant articles.

## Conclusion

This systematic review of reviews summarized the challenges and gaps in the response to outbreaks in Europe in the past 10 years at the healthcare facility level, as highlighted in peer-reviewed literature. All but one of the included articles focused on the COVID-19 pandemic. Most of the reported challenges were at the system level (*n* = 20), followed by staff (*n* = 16), stuff (*n* = 14), and space (*n* = 7).

Key issues included the limited availability of specialized staff, highlighting the need for just-in-time training, peer-to-peer support mechanisms, and improved inter-institutional collaboration. Several studies emphasized the importance of addressing the mental health of HCWs and the complex role of technology in pandemic response, presenting both opportunities and limitations.

We suggest moving beyond crisis mode, employing a whole-of-society approach, a cross-cutting all-hazards approach in combination with a vertical hazard-specific approach.

## Supplementary Information

Below is the link to the electronic supplementary material.

Supplementary Material 1

Supplementary Material 2

## Data Availability

Data are available upon reasonable request.

## Abbreviations

COVID-19: Coronavirus disease 2019
CPR: Cardiopulmonary Resuscitation
EDs: Emergency Departments
GP: General Practitioner
H-EDRM: Health Emergency and Disaster Risk Management
HCWs: Healthcare workers
ICU: Intensive Care Unit
PHC: Primary Healthcare
PICU: Pediatric Intensive Care Unit
PPE: Personal Protective Equipment
PREPSHIELD: Preparedness for Society in Health Crises and Disasters
PRISMA: Preferred Reporting Items for Systematic Reviews and Meta-Analyses
WHO: World Health Organization
