# Supplementary material for: Challenges in healthcare facilities’ response to past outbreaks: a systematic review of reviews
Source: BMC Health Serv Res. 2026 Jan 23;26:141. doi: 10.1186/s12913-025-13934-9 (PMC12849236; doi:10.1186/s12913-025-13934-9)
Supplement: Supplementary file 1 — Supplementary Material 1 [file 12913_2025_13934_MOESM1_ESM.docx]

**Supplemental material 1_Search strings**

**PubMed**

(("epidemic*" [Title/Abstract] OR "pandemic*" [Title/Abstract] OR "health emergency" [Title/Abstract] OR "outbreak*" [Title/Abstract] OR "disease outbreak*" [Title/Abstract] OR "disaster*" [Title/Abstract] OR "crisis*" [Title/Abstract] OR "covid*" [Title/Abstract] OR "COVID-19" [Title/Abstract] OR "SARS-CoV-2" [Title/Abstract] OR "influenza" [Title/Abstract] OR "seasonal flu" [Title/Abstract] OR "seasonal influenza" [Title/Abstract] OR "Mpox" [Title/Abstract] OR "Monkeypox" [Title/Abstract] OR "Monkeypox virus" [Title/Abstract] OR "measles" [Title/Abstract] OR "morbilli" [Title/Abstract] OR "rubeola" [Title/Abstract] OR "red measles" [Title/Abstract] OR "H1N1" [Title/Abstract] OR "A(H1N1)" [Title/Abstract])) AND ("health system*" [Title/Abstract] OR "health facility*" [Title/Abstract] OR "hospital*" [Title/Abstract] OR "ICU" [Title/Abstract] OR "intensive care unit" [Title/Abstract] OR "ED" [Title/Abstract] OR "ER" [Title/Abstract] OR "emergency department*" [Title/Abstract] OR "emergency room*" [Title/Abstract] OR "primary care" [Title/Abstract] OR "PHC" [Title/Abstract] OR "primary health care" [Title/Abstract] OR "primary healthcare" [Title/Abstract]) AND ("response*" [Title/Abstract] OR "effect*" [Title/Abstract] OR "challenge*" [Title/Abstract] OR "gap" [Title/Abstract] OR "difficulty*" [Title/Abstract] OR "impact*" [Title/Abstract] OR "disruption*" [Title/Abstract] OR "discontinuity" [Title/Abstract])

**Scopus**

## ( TITLE-ABS-KEY ( epidemic* OR pandemic* OR "health emergency" OR outbreak* OR "disease outbreak*" OR disaster* OR crisis* OR covid* OR "COVID-19" OR "SARS-CoV-2" OR influenza OR "seasonal flu" OR "seasonal influenza" OR mpox OR "Monkeypox" OR "Monkeypox virus" OR measles OR morbilli OR rubeola OR "red measles" OR h1n1 OR "A(H1N1)" ) AND TITLE-ABS-KEY ( "health system*" OR "health facility*" OR hospital* OR icu OR "intensive care unit" OR ed OR er OR "emergency department*" OR "emergency room*" OR "primary care" OR phc OR "primary health care" OR "primary healthcare" ) AND TITLE-ABS-KEY ( response* OR effect* OR challenge* OR gap* OR difficulty* OR impact* OR disruption* OR discontinuity ) ) AND PUBYEAR > 2008

**WebofScience**

TS=("epidemic*" OR "pandemic*" OR "health emergency" OR "outbreak*" OR "disease outbreak*" OR "disaster*" OR "crisis*" OR "covid*" OR "COVID-19" OR "SARS-CoV-2" OR "influenza" OR "seasonal flu" OR "seasonal influenza" OR "Mpox" OR "Monkey pox" OR "Monkeypox virus" OR "Monkeypox virus" OR "Monkeypox" OR "measles" OR "morbilli" OR "rubeola" OR "red measles" OR "H1N1" OR "A(H1N1)")

AND

(TS=("health system*" OR "health facility*" OR "hospital*" OR "ICU" OR "intensive care unit" OR "ED" OR "ER" OR "emergency department*" OR "emergency room*" OR "primary care" OR "PHC" OR "primary health care" OR "primary healthcare"))

AND

(TS=("response*" OR "effect*" OR "challenge*" OR "gap*" OR "difficulty*" OR "impact*" OR "disruption*" OR "discontinuity"))
