# Supplementary material for: Challenges in healthcare facilities’ response to past outbreaks: a systematic review of reviews
Source: BMC Health Serv Res. 2026 Jan 23;26:141. doi: 10.1186/s12913-025-13934-9 (PMC12849236; doi:10.1186/s12913-025-13934-9)
Supplement: Supplementary file 2 — Supplementary Material 2 [file 12913_2025_13934_MOESM2_ESM.docx]

**Supplemental material 2_** Extraction sheet.

| **General information of the article** | Title |
| --- | --- |
|  | Authors |
|  | Journal |
|  | Publication year |
|  | Number of articles included in the review |
|  | European country/countries where the study was conducted |
| **Study design** | Study period of the review |
|  | Objective of the review |
|  | Type of review |
| **Outbreak** | Epidemic/Pandemic |
|  | Type of epidemic/pandemic |
| **Health facility** | Type of health facility |
|  | Healthcare workers |
|  | Ward |
| **Challenges/Gaps** | Macrodescription |
|  | Detailed description |
|  | Best practices/Lessons learned |
